# *Acinetobacter baumannii* outer membrane protein A induces autophagy in bone marrow‐derived dendritic cells involving the PI3K/mTOR pathway

**DOI:** 10.1002/iid3.830

**Published:** 2023-04-12

**Authors:** Hongyi Tan, Liyan Cao

**Affiliations:** ^1^ Department of Pulmonary and Critical Care Medicine, Huizhou Central People's Hospital Guangdong Medical University Huizhou China; ^2^ Department of Healthcare Associated Affection Management Changsha Central Hospital Changsha China

**Keywords:** *Acinetobacter baumannii*, autophagy, BMDCs, OmpA, PI3K/mTOR pathway

## Abstract

**Background:**

Outer membrane protein A (OmpA) is the major virulence factor of *Acinetobacter baumannii* and plays a wide role in the pathogenesis and antimicrobial resistance of *A. baumannii*. Dendritic cells (DCs) are the most effective antigen‐presenting cells and play a crucial role in regulating the immune response to multiple antigens and immune sentries. We aimed to study the role and molecular mechanisms of OmpA‐induced mouse bone marrow‐derived dendritic cells (BMDCs) autophagy in the immune response of *A. baumannii*.

**Methods:**

First, purified *A. baumannii* OmpA was assessed by sodium dodecyl sulfate‐polyacrylamide gel electrophoresis (SDS‐PAGE) and western blot. OmpA effect on BMDCs viability was evaluated by MTT assay. BMDCs were pretreated with autophagy inhibitor chloroquine or transfected with overexpression plasmids (oe‐NC or oe‐PI3K). Then BMDCs apoptosis, inflammatory cytokines, protein kinase B (PI3K)/mammalian target of rapamycin (mTOR) pathway, and autophagy‐related factors levels were evaluated.

**Results:**

SDS‐PAGE and western blot verified the successful purification of OmpA. BMDCs viability repressed gradually with the increase of OmpA concentration. OmpA treatment of BMDCs led to apoptosis and inflammation in BMDCs. OmpA caused incomplete autophagy in BMDCs, and light chain 3 (LC3), Beclin1, P62, and LC3II/I levels were significantly elevated with the increase of the time and concentration of OmpA treatment. Chloroquine reversed OmpA effects on autophagy in BMDCs, that was, LC3, Beclin1, and LC3II/I levels were reduced, while P62 level was elevated. Furthermore, chloroquine reversed OmpA effects on apoptosis and inflammation in BMDCs. PI3K/mTOR pathway‐related factor expression was affected by OmpA treatment of BMDCs. After overexpression of PI3K, these effects were reversed.

**Conclusions:**

*A. baumannii* OmpA induced autophagy in BMDCs involving the PI3K/mTOR pathway. Our study may provide a novel therapeutic target and theoretical basis for treating infections caused by *A. baumannii*.

## Graphical abstract

Dendritic cells are the most effective antigen‐presenting cells and play a key role in regulating the immune response of multiple antigens and immune sentries. We aimed to study the role and molecular mechanisms of OmpA‐induced mouse bone marrow‐derived dendritic cells autophagy in the immune response of *Acinetobacter baumannii*.

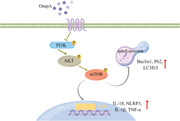

## 1 INTRODUCTION

*Acinetobacter baumannii* is a Gram‐negative bacillus, is also a causative agent of opportunistic infections that commonly occur in immunocompromised, surgical, trauma, or burn patients and could cause infectious disease.^1^, ^2^, ^3^ It is a pressing need to look for novel strategies to control infections caused by *A. baumannii*. Outer membrane protein A (OmpA), a main component of outer membrane protein in Gram‐negative bacteria,^4^ is the main virulence factor of *A. baumannii* and exists in almost all *A. baumannii* strains.^5^ OmpA is important for bacterial adhesion, invasiveness, toxicity, and drug resistance and participates in mitochondrial disassembly and inducing host cell apoptosis.^6^ Therefore, we wanted to investigate the pathogenesis of *A. baumannii* OmpA and its role in antimicrobial resistance.

Dendritic cells (DCs) are the most potent antigen‐presenting cells, serving as initiators of T cell responses to microbial pathogens and playing a key role in regulating immune responses to multiple antigens and immune sentinels.^7^, ^8^ DCs can integrate environmental information and transmit it to other leukocytes, forming adaptive and innate immunity.^9^ DCs could induce immune activation and tolerance in response to peripheral cues.^10^ After microbial infection, DCs become mature. This process is the cornerstone for forming an effective adaptive immune response.^11^ However, the specific regulatory mechanism of *A. baumannii* OmpA in DCs is not fully understood.

Autophagy is a highly conserved cellular degradation process.^12^ Targeting intracellular components for lysosomal degradation by autophagy could maintain cellular homeostasis and counteract external stimuli effects, such as invading pathogens.^13^, ^14^ Serine‐threonine kinase mTOR is a member of the Protein kinase B (PI3K)/AKT/mTOR pathway and is participated in various cellular functions, including proliferation, apoptosis, and autophagy.^15^, ^16^ *A. baumannii* OmpA could promote autophagy in lung cells through the mTOR pathway, ultimately promoting inflammatory mediators release and aggravating host damage.^17^ Furthermore, *Salmonella enterica* serovar Typhimurium OmpA could modulate adaptive immune response via activating DCs and driving Th1 polarization.^18^ However, whether *A. baumannii* OmpA induces DCs damage by regulating autophagy has not been investigated.

Based on the above background, we speculated that *A. baumannii* OmpA might induce autophagy in mouse bone marrow‐derived dendritic cells (BMDCs) through PI3K/mTOR pathway. We intended to investigate the role and molecular mechanism of OmpA‐induced BMDCs autophagy in the immune response of *A. baumannii* at the cellular and molecular level, hoping to clarify the cellular and molecular mechanism of the interaction between *A. baumannii* and BMDCs, and provide a new theoretical basis for treating and preventing *A. baumannii* infection.

## 2 MATERIALS AND METHODS

### 2.1 Acquisition of *A. baumannii* OmpA

OmpA gene (GenBank: 485227) of *A. baumannii* (ATCC 19606) was amplified by polymerase chain reaction (PCR), which contained Xhol and Ndel enzyme cutting sites. PCR product was digested with Xhol and Ndel and ligated with pET 28a (+) to form pET‐28a‐OmpA expression plasmid. Bacterial cells were collected through centrifugation and lysed in binding buffer (0.1% TritionX‐114, 150 mM NaCl, 1 M Tris, pH = 8.0). Cells were ultrasound treated in an ice bath. Cells were centrifuged at 12,000 rpm for 20 min at 4°C. Then the supernatant was collected and purified through Ni‐NTA affinity chromatography. Next, endotoxin was removed from OmpA through gel filtration chromatography (High‐Capacity Endotoxin Removal Resin, Pierce, Thermo Scientific). Then samples were concentrated by ultrafiltration centrifugation (2000 MW cut off, Millipore) and stored at −80°C. Plasmid pET‐28a‐OmpA was purchased from HonroGene. Based on the pET‐28a‐OmpA prokaryotic expression plasmid, prokaryotic protein expression was performed. Identification of OmpA expression was conducted by sodium dodecyl sulfate‐polyacrylamide gel electrophoresis (SDS‐PAGE) and western blot. Membranes were incubated with primary antibody overnight and incubated with secondary antibody. Finally, protein bands were visualized using ECL chemiluminescence solution to observe OmpA purity.

### 2.2 Cell culture and treatment

Mouse BMDCs were purchased from Otwo Biotech (HTX2245C) and grew in DMEM with 10% fetal bovine serum and 1% penicillin/streptomycin. First, BMDCs (1 × 10^6^ cells/mL) were treated with OmpA (0, 5, 10 μg/mL) for 24 h. Further BMDCs were divided into: Control group (BMDCs) and OmpA group (10 μg/mL OmpA treated BMDCs for 24 h).^19^ In addition, 10 μg/mL OmpA treated BMDCs for 6, 12, and 24 h, grouped as: Control, OmpA (6 h), OmpA (12 h), and OmpA (24 h) groups. BMDCs were treated with 5 μg/mL and 10 μg/mL OmpA for 24 h, grouped as: Control, OmpA (5 μg/mL), and OmpA (10 μg/mL) groups. BMDCs were pretreated with autophagy inhibitor chloroquine (10 μM) for 30 min, and BMDCs were treated with 10 μg/mL OmpA for 24 h,^20^ and BMDCs were divided into: Control, OmpA, and OmpA + chloroquine groups. Finally, BMDCs were transfected with overexpression plasmids (oe‐NC or oe‐PI3K) for 48 h, treated with 10 μg/mL OmpA for 24 h and grouped into: OmpA+oe‐NC and OmpA+oe‐PI3K groups. Chloroquine was purchased from MedChemExpress (HY‐17589A). Overexpression PI3K plasmid (oe‐PI3K) and control plasmid (oe‐NC) were purchased from HonroGene. All cell transfections were carried out by Lipofectamine 2000 (11668019, Thermo).

### 2.3 MTT assay

BMDCs were inoculated and cultured, and then added MTT (10 μL, 5 mg/mL, NU679, Dojindo) and incubated for 4 h at 37°C and 5% CO_2_ conditions. Then MTT‐containing medium was removed. Dimethyl sulfoxide (150 μL) was added, shook slowly for 10 min. Finally, absorbance at 490 nm was analyzed using the microplate reader (MB‐530, Heales).

### 2.4 TUNEL

BMDCs were fixed with 3% paraformaldehyde for 40 min at 4°C. 0.1% TritonX‐100 and sodium citrate solution were permeabilized for 5 min at 4°C. Fluorescently labeled nucleotides deoxyuridine triphosphate and terminal deoxynucleotidyl transferase were added to treat BMDCs for 1 h at 37°C. TUNEL‐positive BMDCs were measured by the fluorescence microscope.

### 2.5 Flow cytometry

BMDCs were resuspended in 500 μL 1640 basal medium, and the corresponding antibodies CD11c‐FITC (11‐0114‐82), MHC‐II‐PE (12‐5322‐81), CD86‐PE (12‐0862‐82), and CD80‐PE (12‐0801‐82) provided by eBioscience were added, incubated for 30 min, and set up a negative tube and a single staining tube at the same time. BMDCs were centrifuged at 40 g for 5 min. We discarded supernatant, resuspended BMDCs in 150 μL basal medium, and detected MHC‐II + CD11c, CD80 + CD11c, and CD86 + CD11c double positive levels through flow cytometry (A00‐1‐1102, Beckman).

BMDCs were collected through trypsinization without ethylenediaminetetraacetic acid, and centrifuged at 2000 rpm for 5 min to collect about 3.2 × 10^5^ cells. Binding buffer (500 μL), Annexin V‐APC (5 μL) and propidium iodide (5 μL) were added. BMDCs apoptosis was tested using flow cytometry within 1 h.

### 2.6 Transmission electron microscopy (TEM)

BMDCs were fixed to 2.5% glutaraldehyde for 8 h. Fixative solution was discarded and BMDCs were put into phosphate‐buffered saline buffer, fixed with 1% osmic acid. All levels of ethanol were dehydrated, soaked, embedded in pure epoxy resin, and then baked. Slices were stained with lead citrate, and BMDCs structure was observed by TEM.

### 2.7 Enzyme linked immunosorbent assay (ELISA)

Interleukin‐18 (IL‐18) (AB216165, Abcam), NLRP3 (AB279417, Abcam), IL‐1β (KE10003, Proteintech), and tumor necrosis factor α (TNF‐α) (KE10002, Proteintech) kits were applied to assess IL‐18, NLRP3, IL‐1β, and TNF‐α contents according to the instructions.

### 2.8 Immunofluorescence (IF)

LC3 expression in BMDCs was monitored by IF. Slices were fixed with 4% paraformaldehyde. Then, BMDCs were permeated with 0.5% TritonX‐100 at 37°C for 30 min. BMDCs were blocked with 5% BSA at 37°C for 1 h, and incubated with LC3 (14600‐1‐AP, Proteintech) at 4°C overnight. Dilute fluorescent secondary antibody was added. DAPI (Wellbio) was applied for 10 min at 37°C. Slices were sealed with buffer glycerin and then observed by a fluorescence microscope.

### 2.9 Quantitative real‐time PCR (qRT‐PCR)

Trizol was performed to extract RNA. To reverse transcribe RNA into complementary DNA (cDNA), cDNA reverse transcription kit (#CW2569, CWBIO) was used, and relative expression of genes was examined on a fluorescent quantitative PCR instrument system (QuantStudio1, Thermo) by applying Ultra SYBR Mixture (#CW2601, CWBIO). Genes levels were calculated using the $2^{‐∆∆C_{t}}$ method. β‐actin was acted as reference gene. Primer sequences are as follows: PI3K‐F: GTCCGTCCTGGAGAACTTGG, PI3K‐R: TGAGGCGTTTCTGGATTGC; AKT‐F: CGCCTGCCCTTCTACAACCAG, AKT‐R: GCATGATCTCCTTGGCATCCTC; mTOR‐F: CCGCTACTGTGTCTTGGCAT, mTOR‐R: CAGCTCGCGGATCTCAAAGA; β‐actin‐F: ACATCCGTAAAGACCTCTATGCC, β‐actin‐R: TACTCCTGCTTGCTGATCCAC.

### 2.10 Western blot

Total proteins were isolated by RIPA (P0013B, Beyotime) and subsequently quantified with a BCA kit (BL521A, Biosharp). Total proteins underwent 10% SDS‐PAGE separation and then proteins were transferred to polyvinylidene fluoride membranes. After blocking, membranes were thoroughly mixed overnight with Beclin1 (11306‐1‐AP, 1:1000, Proteintech), P62 (18420‐1‐AP, 1:4000, Proteintech), LC3 (18725‐1‐AP, 1:500, Proteintech), p‐mTOR (ab109268, 1:5000, Abcam), mTOR (ab32028, 1:2000, Abcam), p‐AKT (28731‐1‐AP, 1:3000, Proteintech), AKT (10176‐2‐AP, 1:1000, Proteintech), p‐PI3K (ab278545, 0.5 μg/mL, Abcam), PI3K (ab191606, 1:1000, Abcam), and β‐actin (66009‐1‐Ig, 1:500, Proteintech). HRP‐labeled secondary antibodies were incubated. ECL chemiluminescence solution was used for color development. β‐actin was internal reference.

### 2.11 Statistical analysis

Measurement data were analyzed using Graphpad Prism 8.0 software with mean ± standard deviation as the measure of statistical significance. Student's *t*‐test or one‐way analysis of variance (ANOVA) was applied between two or multiple groups. *p* < .05 was considered statistically significant.

## 3 RESULTS

### 3.1 OmpA induced apoptosis, maturation, and inflammation in BMDCs

**Figure 1:**
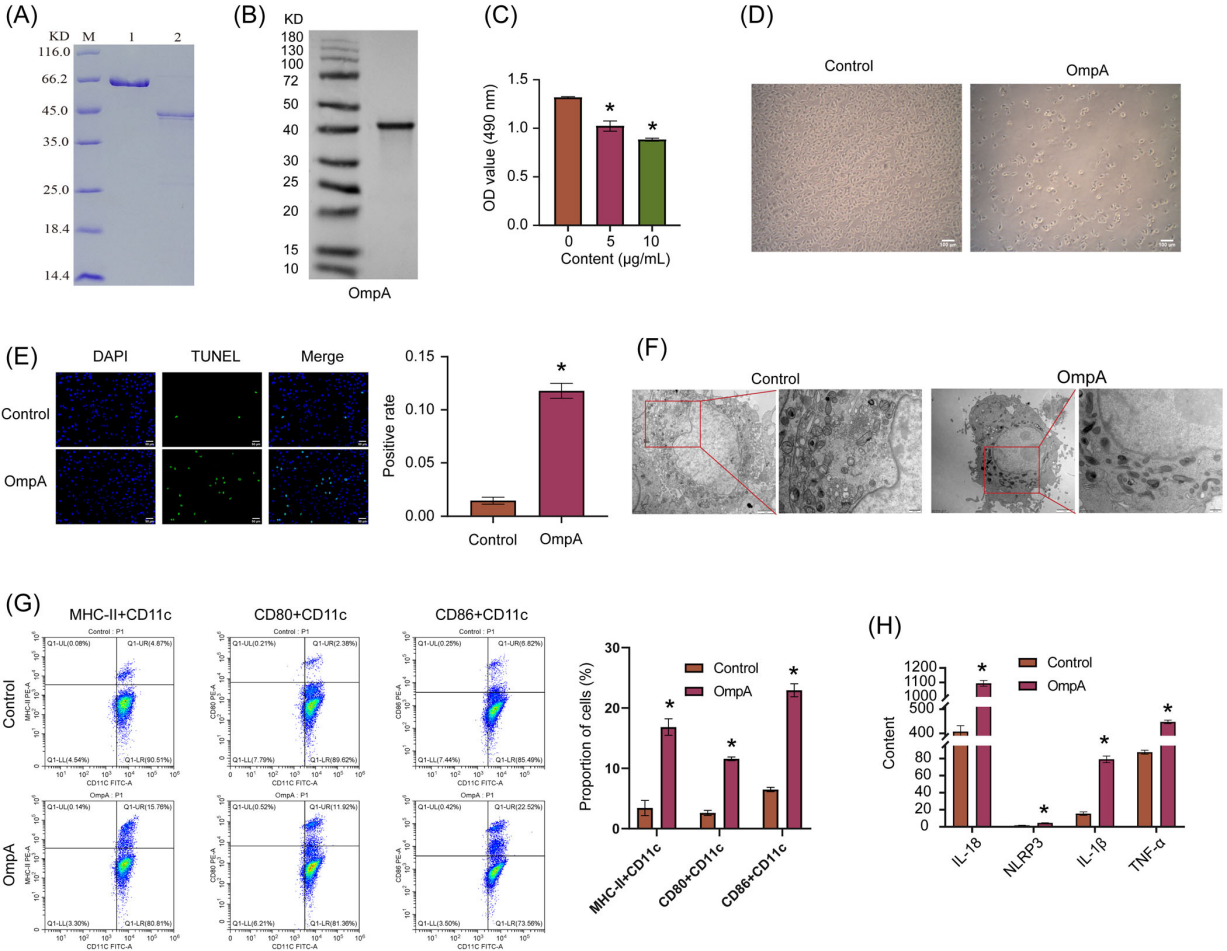
OmpA induced apoptosis, maturation and inflammation in BMDCs. (A) SDS‐PAGE detection of OmpA expression. M: Protein marker; 1: 0.5 mg/mL BSA (66.446 kDa), 2: The purified OmpA (38.41 kDa). (B) Western blot detection of OmpA expression. (C) The effect of different concentrations of OmpA on BMDCs viability measured by MTT. After BMDCs treated with 10 μg/mL OmpA for 24 h, (D) Morphology of BMDCs after BMDCs treated with OmpA (10 μg/mL) for 24 h. (E) BMDCs apoptosis were monitored by TUNEL. Scale bar = 50 μm (×200). (F) The structure of BMDCs was observed by TEM. Scale bar = 2 μm (up)/500 nm (down). (G) The expression of BMDCs mature markers MHC‐II + CD11c, CD80 + CD11c, and CD86 + CD11c were tested by flow cytometry. (H) IL‐18, NLRP3, IL‐1β, TNF‐α contents were monitored using ELISA. *p < .05 versus Control. BMDC, bone marrow‐derived dendritic cell; IL‐18, interleukin‐18; SDS‐PAGE, sodium dodecyl sulfate‐polyacrylamide gel electrophoresis; TEM, transmission electron microscopy.

First, we carried out the purification of *A. baumannii* OmpA, as shown in Figure 1A, SDS‐PAGE verified the successful purification of OmpA. Western blot further verified the expression of OmpA (Figure 1B). After treatment of BMDCs with different concentrations of OmpA, MTT assay showed BMDCs viability repressed gradually with the increase of OmpA concentration (Figure 1C). Figure 1D showed the morphological figure of BMDCs, and BMDCs number in the OmpA group was significantly reduced. Compared with the control group, BMDCs apoptosis in the OmpA group was also significantly promoted (Figure 1E). TEM results revealed cells in the control group were larger, with no condensed cytoplasm, a relatively complete organelle structure, a complete nuclear membrane, and a small part of chromatin condensation. The cells in the OmpA group were shrunk, the cytoplasm was condensed and deepened, other organelle structures were relatively intact, the nuclear membrane double membrane was intact, the chromatin was condensed around the nuclear membrane, the layers were crescent‐shaped or irregular, and there were large areas of chromatin condensation (Figure 1F). In addition, BMDCs mature markers MHC‐II + CD11c, CD80 + CD11c, and CD86 + CD11c double positive levels were elevated in the OmpA group compared with the Control group (Figure 1G). Moreover, IL‐18, NLRP3, IL‐1β, and TNF‐α contents were elevated in the OmpA group than Control group (Figure 1H). Collectively, OmpA might lead to apoptosis, maturation, and inflammation in BMDCs.

### 3.2 OmpA affected autophagy in BMDCs

**Figure 2:**
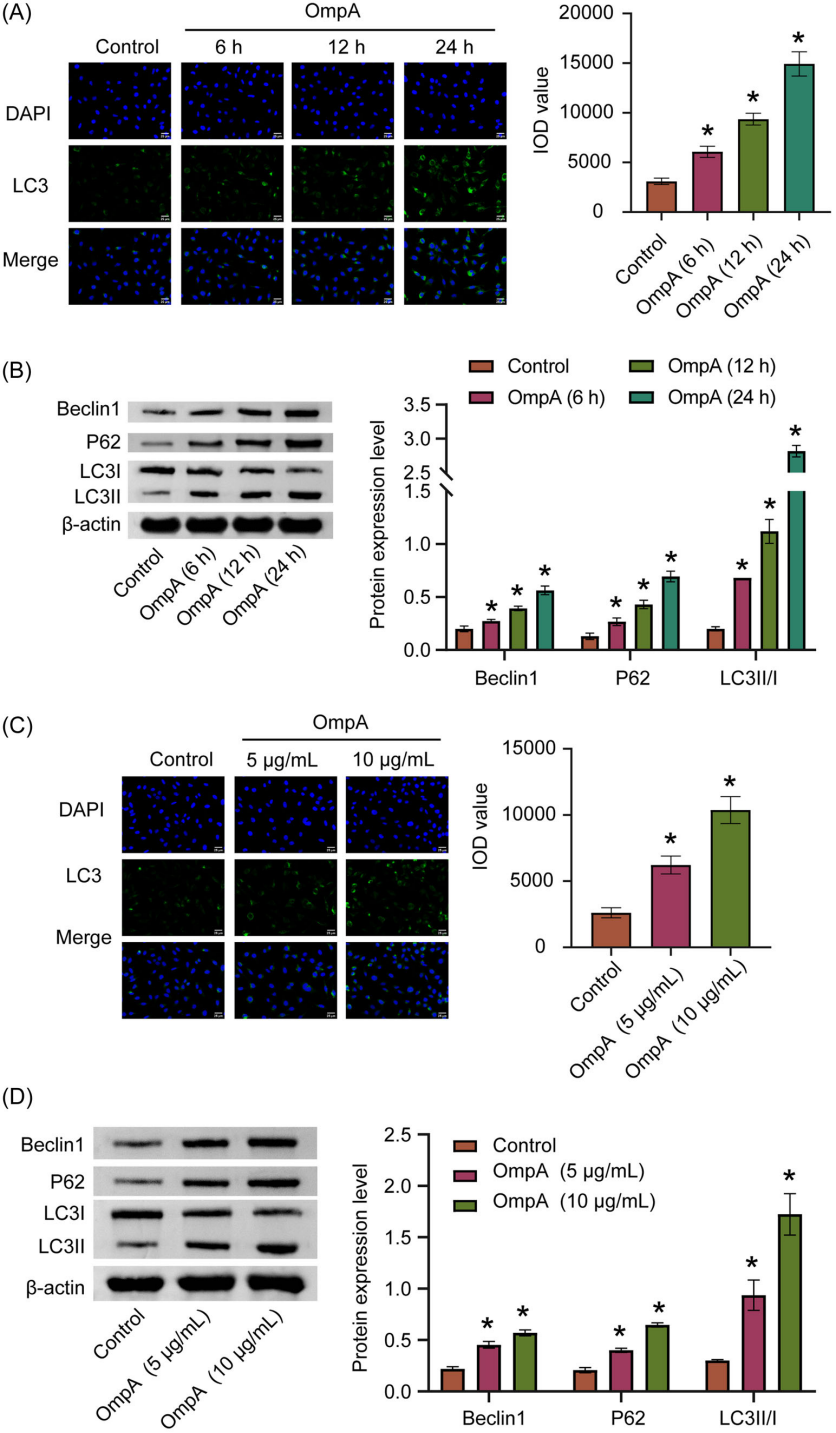
OmpA affected autophagy in BMDCs. After BMDCs were treated with OmpA (10 μg/mL) for 6, 12, and 24 h (A). IF was applied to assess LC3 expression. Scale bar = 25 μm (×400). (B) Western blot detection of Beclin1, P62, and LC3II/I expressions. After BMDCs were treated with OmpA (5 μg/mL and 10 μg/mL) for 24 h (C). LC3 expression was tested using IF. Scale bar = 25 μm (×400). (D) Western blot detection of Beclin1, P62, and LC3II/I expressions. *p < .05 versus Control. BMDC, bone marrow‐derived dendritic cell; DAPI, 4′,6‐diamidino‐2‐phenylindole; LC, light chain; TNF, tumor necrosis factor.

To investigate whether OmpA affected autophagy in BMDCs, we examined whether autophagy markers changed with the time and concentration of OmpA treatment, respectively. After BMDCs were treated with OmpA (10 μg/mL) for 6, 12, and 24 h, LC3, Beclin1, P62, and LC3II/I levels were elevated with the increase of treatment time (Figure 2A,B). After BMDCs were treated with OmpA (5 and 10 μg/mL) for 24 h, LC3, Beclin1, P62, and LC3II/I levels were significantly promoted with increasing treatment concentrations (Figures 2C and 2D). These results indicated OmpA caused incomplete autophagy in BMDCs, leading to the conversion of LC3I to LC3II, while inhibiting P62 degradation.

### 3.3 OmpA promoted the maturation and inflammation of BMDCs by affecting autophagy

**Figure 3:**
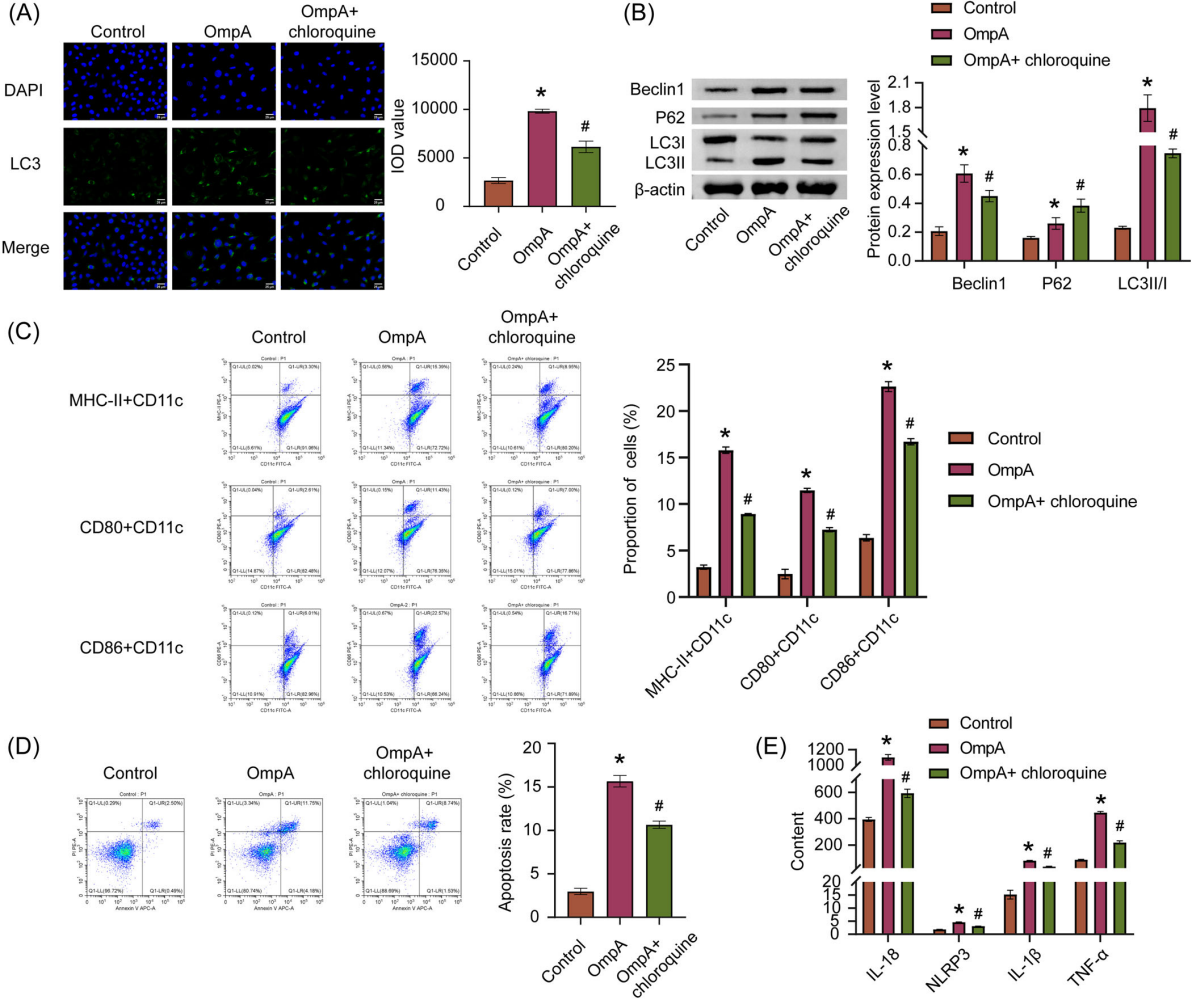
OmpA promoted the maturation and inflammation of BMDCs by affecting autophagy. BMDCs were pretreated with chloroquine for 30 min, and BMDCs were treated with OmpA (10 μg/mL) for 24 h. (A) IF was utilized to assess LC3 level. Scale bar = 25 μm (×400). (B) Western blot detection of Beclin1, P62, and LC3II/I expressions. (C) The levels of MHC‐II + CD11c, CD80 + CD11c, CD86 + CD11c, the mature markers of BMDCs, were monitored by flow cytometry. (D) Flow cytometry assessment of BMDCs apoptosis. (E) IL‐18, NLRP3, IL‐1β, and TNF‐α contents were tested using ELISA. *p < .05 versus Control, ^#^ *p* < .05 versus OmpA. BMDC, bone marrow‐derived dendritic cell; LC, light chain; IL, interleukin; TNF, tumor necrosis factor.

Next, we pretreated BMDCs with chloroquine, an autophagy inhibitor, and treated BMDCs with OmpA (10 μg/mL) for 24 h. Chloroquine reversed OmpA effects on autophagy in BMDCs, that was, LC3, Beclin1, and LC3II/I levels were suppressed, while P62 level was upregulated (Figure 3A,B). Flow cytometry was performed to further examine maturation markers levels in BMDCs. Compared with the control group, MHC‐II + CD11c, CD80 + CD11c, and CD86 + CD11c double positive levels were promoted in the OmpA group, while chloroquine reversed the effects of OmpA on MHC‐II + CD11c, CD80 + CD11c, and CD86 + CD11c double positive levels (Figure 3C). Furthermore, chloroquine reversed the effects of OmpA on apoptosis and inflammation in BMDCs. That meant BMDCs apoptosis was inhibited, and IL‐18, NLRP3, IL‐1β, and TNF‐α contents were also repressed (Figure 3D,E). Taken together, OmpA promoted the maturation and inflammation of BMDCs by affecting autophagy.

### 3.4 OmpA affected PI3K/mTOR pathway

**Figure 4:**
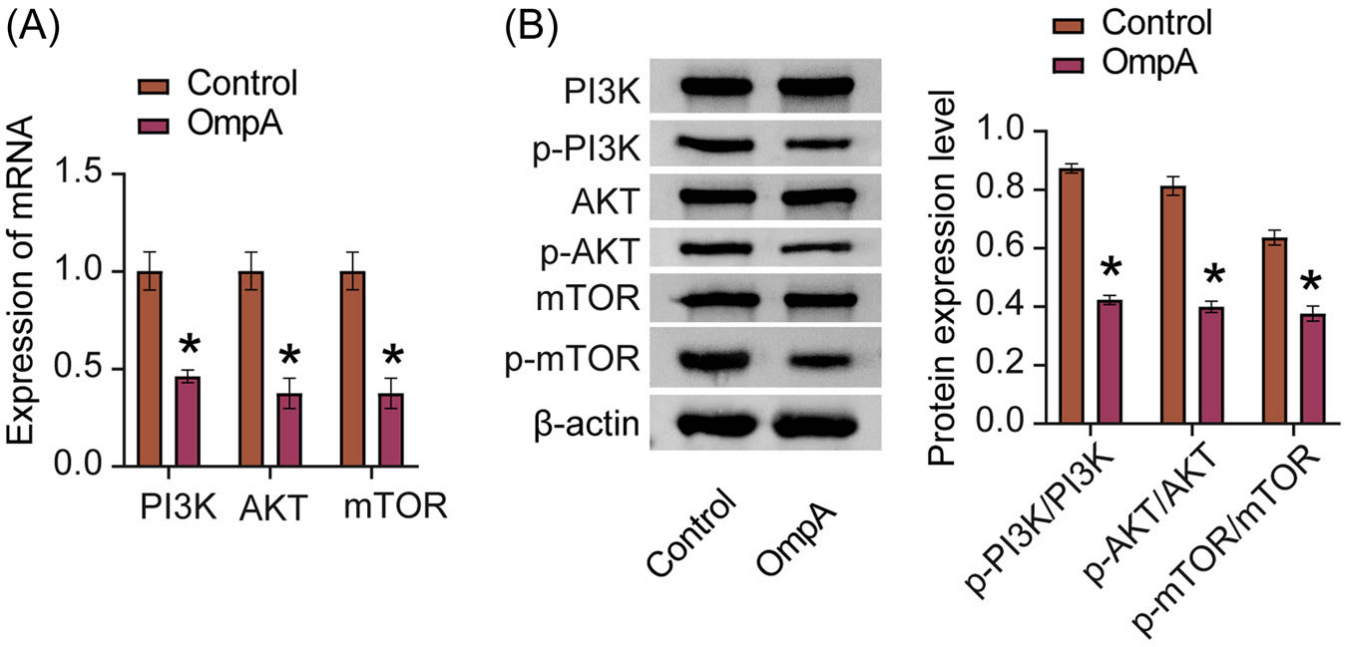
OmpA affected PI3K/mTOR pathway. After BMDCs were treated with OmpA (10 μg/mL) for 24 h. (A) qRT‐PCR assessment of PI3K, AKT and mTOR expressions. (B) Western blot detection of PI3K/mTOR pathway‐related proteins expressions. *p < .05 versus Control. BMDC, bone marrow‐derived dendritic cell; PI3K/AKT, protein kinase B; mTOR, mammalian target of rapamycin.

To explore whether OmpA affected PI3K/mTOR pathway, we next assessed PI3K/mTOR pathway‐related factors expressions. After BMDCs were treated with OmpA, PI3K, AKT, and mTOR messenger RNA expressions were significantly repressed, p‐PI3K/PI3K, p‐AKT/AKT, and p‐mTOR/mTOR protein expressions were also significantly suppressed (Figure 4A,B). This demonstrated that OmpA might affect PI3K/mTOR pathway.

### 3.5 BMDCs autophagy was reduced after overexpression of PI3K

**Figure 5:**
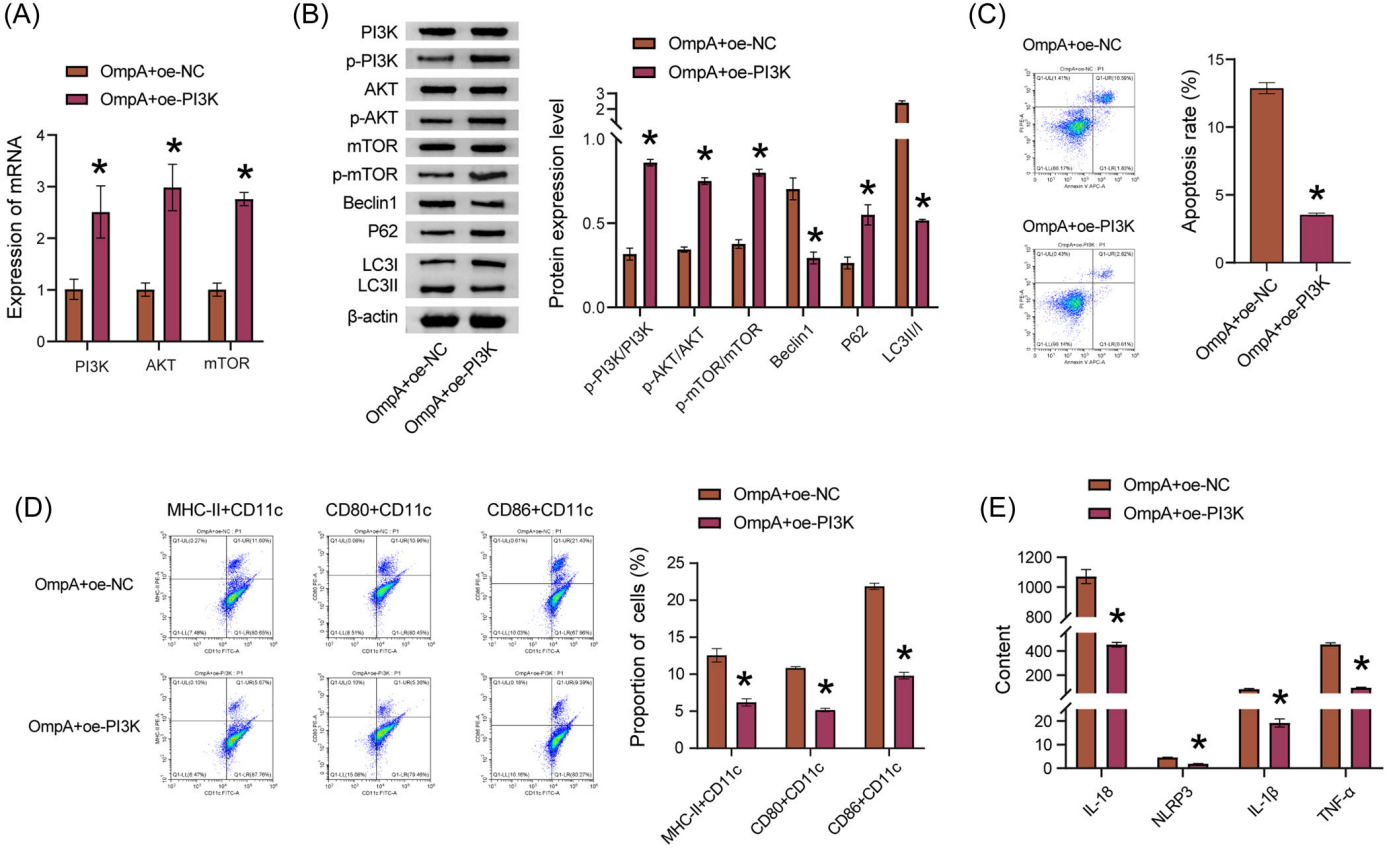
BMDCs autophagy was reduced after overexpression of PI3K. The overexpression plasmids (oe‐NC or oe‐PI3K) were first transfected, and BMDCs were treated with OmpA (10 μg/mL) for 24 h. (A) PI3K, AKT and mTOR expressions were monitored using qRT‐PCR. (B) Western blot detection of PI3K/mTOR pathway related proteins, Beclin1, P62, and LC3II/I expressions. (C) Flow cytometry detection of BMDCs apoptosis. (D) The expression of BMDCs mature markers MHC‐II + CD11c, CD80 + CD11c, and CD86 + CD11c were tested by flow cytometry. E. IL‐18, NLRP3, IL‐1β, and TNF‐α contents were assessed through ELISA. *p < .05 versus OmpA+oe‐NC. BMDC, bone marrow‐derived dendritic cell; IL, interleukin; PI3K/AKT, protein kinase B; mTOR, mammalian target of rapamycin; TNF, tumor necrosis factor.

Finally, we overexpressed PI3K. Compared with the OmpA+oe‐NC group, PI3K, AKT, mTOR mRNA levels in OmpA+oe‐PI3K group were significantly elevated, and p‐PI3K/PI3K, p‐AKT/AKT, p‐mTOR/mTOR, and P62 expressions were also significantly promoted. However, Beclin1 and LC3II/I levels were repressed (Figure 5A,B). In addition, after overexpression of PI3K, BMDCs apoptosis was reduced, MHC‐II + CD11c, CD80 + CD11c, CD86 + CD11c double positive levels, IL‐18, NLRP3, IL‐1β, and TNF‐α contents were also repressed (Figure 5C–E). These results indicated that BMDCs autophagy was reduced after overexpression of PI3K.

## 4 DISCUSSION

*A. baumannii* is an opportunistic pathogen and a major cause of healthcare‐associated infections.^21^ The rapid development of new biological properties under external stress complicates the pathogenesis. In this study, OmpA on the outer membrane of *A. baumannii* was taken as the key research object. We focused on OmpA‐induced autophagy in BMDCs and its inhibition of autophagy to clarify the immune regulation of OmpA in BMDCs. Our research showed that in the *A. baumannii*‐induced immune response process, OmpA could induce autophagy in DCs, which involved the PI3K/mTOR pathway.

OmpA is a β‐barrel integral membrane protein located in the bacterial outer membrane.^22^ OmpA plays a broad role in the pathogenesis and antimicrobial resistance of *A. baumannii*.^23^ Therefore, OmpA is an innovative target for antivirulence therapy against *A. baumannii*. A previous study reported that OmpA exacerbated *A. baumannii* lung inflammation by inhibiting caspase‐1 degradation and modulating NLRP3 inflammasome activation.^24^ OmpA‐deficient *A. baumannii* outer membrane vesicles elicit reduced inflammatory responses.^25^ Lee et al. reported *A. baumannii* OmpA induced early‐onset apoptosis and late‐onset necrosis in DCs.^26^ Consistent with these studies, we revealed that OmpA treatment of BMDCs resulted in apoptosis and inflammation in BMDCs.

DCs are the most important antigen‐presenting cells known and act as a bridge between innate and adaptive immune responses, and effective T‐cell immune responses depend on the maturation and activation of DCs.^10^, ^27^ Autophagy, a key pathway of cellular homeostasis, is active in DCs and upregulated in various inflammatory conditions.^28^ During viral infection, autophagy is triggered in immune cells. In macrophages and DCs, the goal is to expose virion‐derived fragments to prime lymphocytes and initiate immune responses.^29^ In the process of innate immunity in the face of foreign microorganisms, autophagy directly or indirectly participates in the maturation, migration, activation of T cells and cytokine secretion of DCs.^30^ *A. baumannii* OmpA is related to autophagy, which is vital in its pathogenicity.^17^ Our study showed that OmpA affected autophagy in BMDCs. Lee et al. found that high concentrations of OmpA (80 nM) could lead to apoptosis of DCs. In comparison, low concentrations of OmpA (5 nM) could lead to DCs maturation, manifested by the elevated expression of stimulatory molecules CD80, CD86, and MHC‐II and lead to a Th1‐type immune response.^18^ This demonstrated that OmpA could cause the maturation of DCs. Consistent with our study, OmpA promoted the maturation and inflammation of BMDCs by affecting autophagy.

PI3K/mTOR pathway regulates key cellular processes, including cell growth, metabolism, and autophagy.^31^ OmpA could induce autophagy, but OmpA prevents fusion of autophagosome and lysosome and interferes with the activation of autophagy, resulting in incomplete autophagy. OmpA regulates the process of autophagy is achieved through the JNK signaling pathway.^19^ Our study demonstrated that OmpA might help *A. baumannii* escape the host immune attack and survive by causing incomplete autophagy of BMDCs, eventually leading to the colonization or persistent infection of *A. baumannii* in the body, which involves the PI3K/mTOR pathway.

However, there are some limitations in this study. Due to time and funding constraints, we cannot add more cell lines and explore the mechanisms in animals. In the future, with sufficient time and funding, we will validate our conclusions in human BMDCs and further explore the possible mechanisms in depth in animals.

In conclusion, our study confirmed that OmpA could induce autophagy but incomplete autophagy in BMDCs. Furthermore, OmpA was an important virulence factor of *A. baumannii*, which was related to the incomplete autophagy of BMDCs caused by *A. baumannii*. It was a key molecule that helped *A. baumannii* escape the immune response. It was expected that OmpA could be used as a new therapeutic target for *A. baumannii* infection.

## AUTHOR CONTRIBUTIONS

**Hongyi Tan**: Formal analysis; Investigation; Software; Validation; Visualization; Writing—original draft; Writing—review & editing. **Liyan Cao**: Conceptualization; Data curation; Formal analysis; Investigation; Methodology; Resources; Software; Validation; Writing—review & editing.

## CONFLICT OF INTEREST STATEMENT

The authors declare no conflict of interest.

## Data Availability

All data included in this study are available upon request by contacting the first author or corresponding author.
